# Refining the evolutionary tree of the horse Y chromosome

**DOI:** 10.1038/s41598-023-35539-0

**Published:** 2023-06-02

**Authors:** Elif Bozlak, Lara Radovic, Viktoria Remer, Doris Rigler, Lucy Allen, Gottfried Brem, Gabrielle Stalder, Caitlin Castaneda, Gus Cothran, Terje Raudsepp, Yu Okuda, Kyaw Kyaw Moe, Hla Hla Moe, Bounthavone Kounnavongsa, Soukanh Keonouchanh, Nguyen Huu Van, Van Hai Vu, Manoj Kumar Shah, Masahide Nishibori, Polat Kazymbet, Meirat Bakhtin, Asankadyr Zhunushov, Ripon Chandra Paul, Bumbein Dashnyam, Ken Nozawa, Saria Almarzook, Gudrun A. Brockmann, Monika Reissmann, Douglas F. Antczak, Donald C. Miller, Raheleh Sadeghi, Ines von Butler-Wemken, Nikos Kostaras, Haige Han, Dugarjaviin Manglai, Abdugani Abdurasulov, Boldbaatar Sukhbaatar, Katarzyna Ropka-Molik, Monika Stefaniuk-Szmukier, Maria Susana Lopes, Artur da Câmara Machado, Valery V. Kalashnikov, Liliya Kalinkova, Alexander M. Zaitev, Miguel Novoa‐Bravo, Gabriella Lindgren, Samantha Brooks, Laura Patterson Rosa, Ludovic Orlando, Rytis Juras, Tetsuo Kunieda, Barbara Wallner

**Affiliations:** 1 Institute of Animal Breeding and Genetics, University of Veterinary Medicine Vienna, 1210 Vienna, Austria; 2 Vienna Graduate School of Population Genetics, University of Veterinary Medicine Vienna, 1210 Vienna, Austria; 3 Research Institute of Wildlife Ecology, University of Veterinary Medicine Vienna, 1210 Vienna, Austria; 4 School of Veterinary Medicine and Biomedical Sciences, Texas A&M University, College Station, TX 77843 USA; 5 Museum of Dinosaur Research, Okayama University of Science, Okayama, Japan; 6 Department of Pathology and Microbiology, University of Veterinary Science, Yezin, Nay Pyi Taw 05282 Myanmar; 7 Department of Genetics and Animal Breeding, University of Veterinary Science, Yezin, Nay Pyi Taw 05282 Myanmar; 8 National Agriculture and Forestry Research Institute (Lao) Resources, Livestock Research Center, Xaythany District, Vientiane, Laos; 9 Faculty of Animal Science and Veterinary Medicine, University of Agriculture and Forestry, Hue University, Hue, Vietnam; 10 Faculty of Animal Science, Veterinary Science and Fisheries, Agriculture and Forestry University, Rampur, 44209 Nepal; 11 Graduate School of Integrated Sciences for Life, Hiroshima University, Higashi-Hiroshima, 739-8528 Japan; 12 Radiobiological Research Institute, JSC Astana Medical University, Astana, 010000 Republic of Kazakhstan; 13 Institute of Biotechnology, National Academy of Sciences of the Kyrgyz Republic, Bishkek, 720071 Kyrgyz Republic; 14 Graduate School of Environmental and Life Science, Okayama University, Okayama, Japan; 15 Faculty of Animal Science and Veterinary Medicine, Patuakhali Science and Technology University, Barishal, Bangladesh; 16 Institute of Biological Sciences, Mongolian Academy of Sciences, Ulaan Baator, Mongolia; 17 Primate Research Institute, Kyoto University, Aichi, Japan; 18 Albrecht Daniel Thaer-Institut, Humboldt-Universität zu Berlin, 10115 Berlin, Germany; 19 Baker Institute for Animal Health, College of Veterinary Medicine, Cornell University, Ithaca, NY 14853 USA; 20 Barb Horse Breeding Organisation VFZB E. V., Verein der Freunde und Züchter Des Berberpferdes E.V., Kirchgasse 11, 67718 Schmalenberg, Germany; 21 Amaltheia, Argirokastrou 51, 15669 Papagou, Greece; 22 Inner Mongolia Key Laboratory of Equine Genetics, Breeding and Reproduction, College of Animal Science, Equine Research Center, Inner Mongolia Agricultural University, Hohhot, 010018 China; 23 Department of Agriculture, Faculty of Natural Sciences and Geography, Osh State University, 723500 Osh, Kyrgyzstan; 24 Sector of Surveillance and Diagnosis of Infectious Diseases, State Central Veterinary Laboratory, Ulaanbaatar, 17024 Mongolia; 25 National Research Institute of Animal Production, Animal Molecular Biology, 31-047 Cracow, Poland; 26 Biotechnology Centre of Azores, University of Azores, 9700-042 Angra do Heroísmo, Portugal; 27 All-Russian Research Institute for Horse Breeding, Ryazan, 391105 Russia; 28 Genética Animal de Colombia SAS., Av. Calle 26 #69-76, 111071 Bogotá, Colombia; 29 Department of Animal Breeding and Genetics, Swedish University of Agricultural Sciences, 75007 Uppsala, Sweden; 30 Department of Biosystems, Center for Animal Breeding and Genetics, KU Leuven, 3001 Leuven, Belgium; 31 Department of Animal Science, UF Genetics Institute, University of Florida, Gainesville, FL 32610 USA; 32 Department of Agriculture and Industry, Sul Ross State University, Alpine, TX 79832 USA; 33 Centre d’Anthropobiologie et de Génomique de Toulouse, Université Paul Sabatier, Toulouse, France; 34 Faculty of Veterinary Medicine, Okayama University of Science, Imabari, Japan

**Keywords:** Evolutionary genetics, Population genetics, Genetic variation

## Abstract

The Y chromosome carries information about the demography of paternal lineages, and thus, can prove invaluable for retracing both the evolutionary trajectory of wild animals and the breeding history of domesticates. In horses, the Y chromosome shows a limited, but highly informative, sequence diversity, supporting the increasing breeding influence of Oriental lineages during the last 1500 years. Here, we augment the primary horse Y-phylogeny, which is currently mainly based on modern horse breeds of economic interest, with haplotypes (HT) segregating in remote horse populations around the world. We analyze target enriched sequencing data of 5 Mb of the Y chromosome from 76 domestic males, together with 89 whole genome sequenced domestic males and five Przewalski’s horses from previous studies. The resulting phylogeny comprises 153 HTs defined by 2966 variants and offers unprecedented resolution into the history of horse paternal lineages. It reveals the presence of a remarkable number of previously unknown haplogroups in Mongolian horses and insular populations. Phylogenetic placement of HTs retrieved from 163 archaeological specimens further indicates that most of the present-day Y-chromosomal variation evolved after the domestication process that started around 4200 years ago in the Western Eurasian steppes. Our comprehensive phylogeny significantly reduces ascertainment bias and constitutes a robust evolutionary framework for analyzing horse population dynamics and diversity.

## Introduction

Sequence variation on the male specific region of the mammalian Y chromosome (MSY) reflects the demographic history of the male lineages in a population. The potency of the MSY results from its unique inheritance mechanism, which is strictly paternal without recombination. It retains sequential records of the accumulation of genetic diversity within a lineage through time and, thus, MSY phylogenetic reconstruction can disclose the order of descent of haplotypes (HT) or haplogroups (HG)^1^. This allows for the study of the level of structure among assessed populations in fine detail. MSY variation is most extensively studied in humans, in which it has been characterized in detail over the past two decades. In humans, MSY studies have revealed multiple episodes of past demographic expansions and contractions and recent and ancient migrations^2–4^. Furthermore, MSY analysis has become an invaluable resource for genealogical and forensic analyses^5,6^.

Compared to humans, our knowledge of MSY variation in domestic animals is still rudimentary. This is because the complex structure makes the Y chromosome difficult to assemble and sequence^7^. Recent improvements in the production and analysis of high-throughput sequencing data have resulted in the release of Y phylogenies for several domestic animals, for example cattle^8,9^, sheep^10,11^, goats^12,13^, Bactrian camels^14^ and dogs^15^. Many of those studies highlight bursts of expansion of male lineages in the past few thousand years, mostly following the rapid amplification of specific lineages after domestication and as the result of intensive reproductive bias in modern breeding.

The MSY variation in horses has also received scientific attention. Long assumed as a diversity wasteland^16^, horse MSY information content was recently improved thanks to the steady progress from several research groups. This involved the assembly of MSY reference contigs as well as the production of resequencing data from modern and ancient horses and comprehensive genotyping. It was shown that the Y chromosome diversity was pronounced in wild horses and early domesticates^17,18^, but steadily decreased after domestication^19^, especially in the past 300 years, consistent with the diversity drops observed in autosomes^20^. Stallion focused selection has mainly provided the main breeding scheme over hundreds of years of reproductive management^21,22^, which makes the detailed resolution of horse MSY trajectories particularly instructive for reconstructing the domestication and breeding history. The definition of a stable, comprehensive, and temporally calibrated HT phylogeny represents a crucial prerequisite towards this objective.

The current horse MSY topology is based on 2226 variants ascertained from NGS data mapped to the 5.8 Mb single copy Y regions represented in the LipY764 assembly^23,24^. In this phylogeny, the HTs segregating in modern domesticates and Przewalski’s horses appear clearly separated and a HG present in only a few Asian horses branches off early from the domestic clade^25^. Most of the Y HTs present in modern domesticates cluster together within a major clade, with the majority of breeds studied to date forming the so-called ‘Crown’ HG. The monophyletic Crown group is now widely distributed and originated approximately 1500 years ago^23^; the Crown is generally considered as a hallmark of the exceptional incorporation of Oriental stallions in breeding programs during the last hundred years^26^, including through the expansion of massively influential refined breeds, such as the Arabian and Thoroughbred horses^23,24,27^. So far, the domestic horses’ Y tree only includes six branches outside the Crown group, two of which originate in Northern Europe (N and I), and four of Asian origin (O, J, M and Y)^25^. However, the ascertainment of polymorphisms was so far mainly based on the variation present in modern breeds of commercial interest^23,24^. Local landraces, especially in Asia, have been mostly overlooked despite being known to have retained private MSY lineages^24,28–30^. Therefore, the current MSY marker panel does not provide sufficient resolution to clearly ascertain the horse Y chromosome history.

In this study, we have carried out a comprehensive Y chromosome genotyping of the populations that presumably harbor private HTs, with the aim to extend the characterization of the horse MSY variation and reduce the ascertainment bias in the MSY marker panel by target enriched MSY sequencing. As a result, we present an improved horse MSY phylogeny encompassing previously uncharacterized major HGs. We further date major branching points by leveraging an extensive panel of ancient horse samples previously sequenced. Finally, we illustrate the potential of our new MSY phylogeny to trace the paternal origin and development of domestic horse populations in history.

## Results and discussion

### Reporting MSY haplogroups in horse populations around the globe

**Figure 1:**
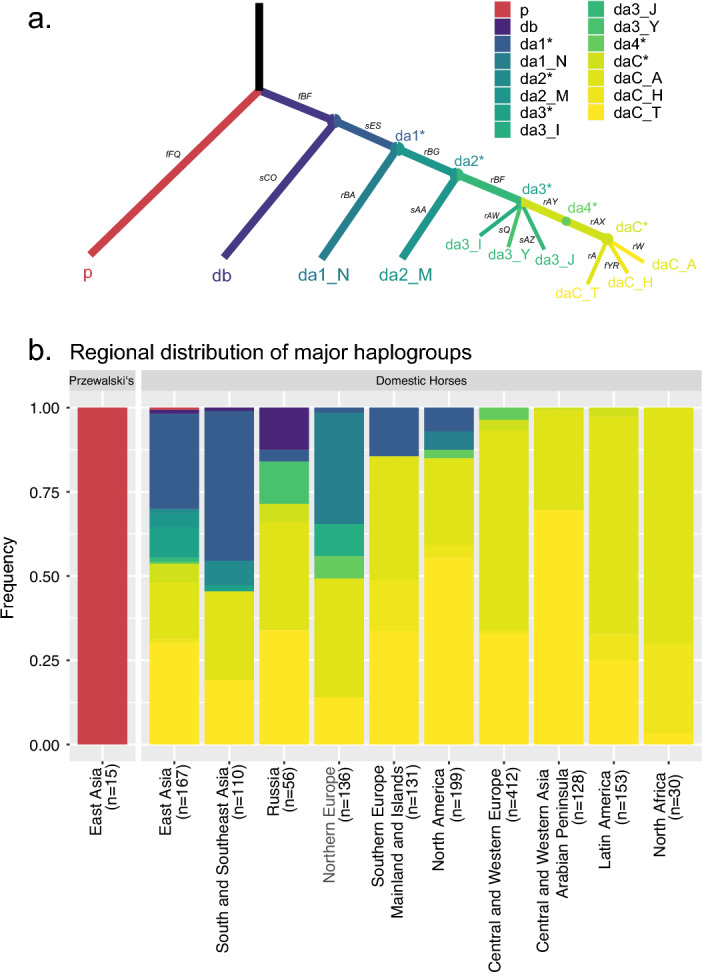
Distribution of MSY haplogroups across horse populations. (**a**) The tree shows the topology of ten mjHGs resulting from the 16 MSY markers (Supplementary Table S1) selected for genotyping. The markers are given on branches. The five observed inner node clustering positions are denoted by a ‘*’ in their nomenclature. (**b**) MSY HG frequencies from genotyping 15 Przewalski’s horses and 1522 domestic horses representing 135 breeds. Domestic horses were grouped into 10 geographic regions and the number of samples from each region is given in parenthesis. The color code is according to (**a**) and the full dataset is given in Supplementary Table S2.

A previously-released fine-scale MSY HT topology^23,24^ provided the basis for the selection of 16 MSY diagnostic markers for major haplogroups (mjHGs) in modern horse breeds. Genotyping these markers helped to differentiate Przewalski’s horses’, as well as six non-Crown and three Crown mjHGs (Fig. 1a, Supplementary Table S1). In order to test whether other non-Crown HGs could be identified, we genotyped those loci amongst 15 additional Przewalski’s horses as well as 1522 domestic horses representing 135 breeds. These breeds spanned a broad geographical and phenotypic distribution range, and not only included many economically important and globally distributed breeds, but also multiple breeds with a documented history of genetic isolation, such as Icelandic horses, or horse populations raised in Mongolia, Yakutia and Japan (dataset given in Supplementary Table S2). The previously established HG nomenclature^23,24,26^ was adapted to reflect the newly generated data in an unambiguous naming system (Supplementary Information File S1).

Genotyping results corroborated the pronounced signature of Crown lineages (indicated as daC_A/H/T in Fig. 1b) across all geographical regions, with Crown HG reaching frequencies from 48% in South East Asia, to full fixation in Central and Western Asia, the Arabian Peninsula, North Africa, and Latin America (Fig. 1b, Supplementary Table S2). Our comprehensive MSY panel further unveiled the distribution of previously defined non-Crown HGs around the world. For example, we confirmed the presence of HGs db, da2_M, da3_J, da3_Y in Asia and/or Russia. Interestingly, HGs da1_N and da1_I were detected in Northern European breeds, but da1_N was identified in North American populations for the first time. Similarly, HG p was confirmed to be common amongst Przewalski’s horses, but was also in a single Mongolian horse.

Strikingly, our dataset included 302 non-Crown samples, 191 of which clustered outside of the mjHGs previously defined. These samples specifically placed on the inner nodes of the MSY phylogeny, as indicated with a ‘*’ suffix (i. e. da1*, da2*, da3*, and da4*), most frequently along the da1* inner node (N = 133). Together with 30 samples allocated to the basal Crown node (daC*), our results uncovered a considerable fraction of previously unknown Y chromosomal diversity in domestic horses. This motivated the further characterization of those HTs using next-generation sequencing data and a finer-grain horse MSY phylogeny.

### Improving the horse MSY tree

**Figure 2:**
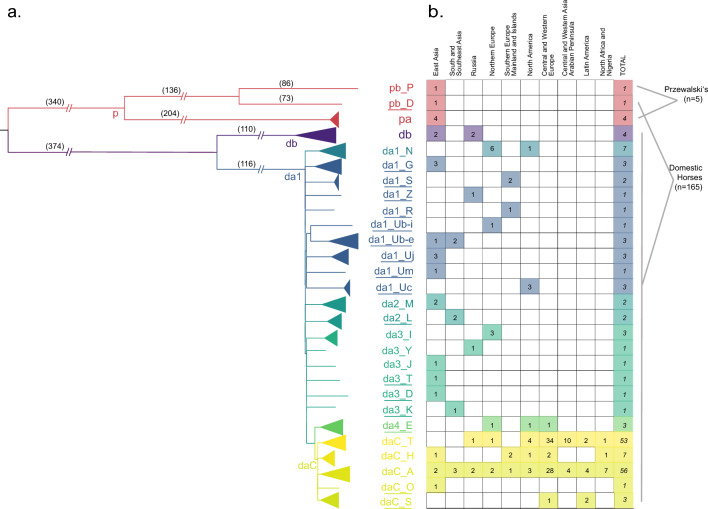
Horse Y phylogeny from stringently filtered SNPs. (**a**) The maximum parsimony tree is based on 2678 SNPs gathered from NGS data analysis of 165 domestic horse and 5 Przewalski’s horse samples. Number of mutations are given on the branches of the tree. Haplogroups newly identified in this study are marked with an underline. Because of the extensive number of samples in Crown haplogroup, it is collapsed into its five major branches. (**b**) Shows the number of samples in each cluster and their region of origin.

We used the genotyping data to select 31 samples clustering on the inner nodes of the MSY phylogeny and the Mongolian horse in HG p for target enriched sequencing (TES) of 5.06 Mb of MSY single copy Y (scY) region. These were processed together with an additional set of 45 samples representing haplogroups or breeds hitherto overlooked. TES data were analyzed together with samples previously characterized through TES experiments (N = 39,^24^) and whole-genome sequencing (WGS) (N = 55)^23^, representing a final dataset of 165 domestic horses and five Przewalski’s horses (see Supplementary Table S3). Variant calling and stringent filtering provided a total of 2678 Single Nucleotide Polymorphisms (SNPs) that were used for maximum parsimony tree reconstruction (Fig. 2a, Supplementary Fig. S1).

The resulting finer-grained phylogeny confirmed the general separation of domestic (da/db) and Przewalski’s horse’s (pa/pb) HGs^23,26^, in line with mjHGs diagnostic markers, as well as the Mongolian horse forming a new branch within Przewalski’s group (pb_D). All other domestic horses grouped into clades da or db and the HTs in da showed a pronounced multifurcation from the basal node da1 (Fig. 2). In total, we identified 21 non-Crown and 5 Crown mjHGs in domestic horses, 17 of which were defined for the first time (Fig. 2, underlined labels) and mainly comprised Asian horses or isolated populations. In the Crown, we defined two novel basal branching HGs; daC_O in a single sample from Mongolia, and daC_S in one European and two Latin American horses. Finally, the Crown mjHG daC_A was represented by the largest number of samples (N = 56) and showed a worldwide distribution.

**Figure 3:**
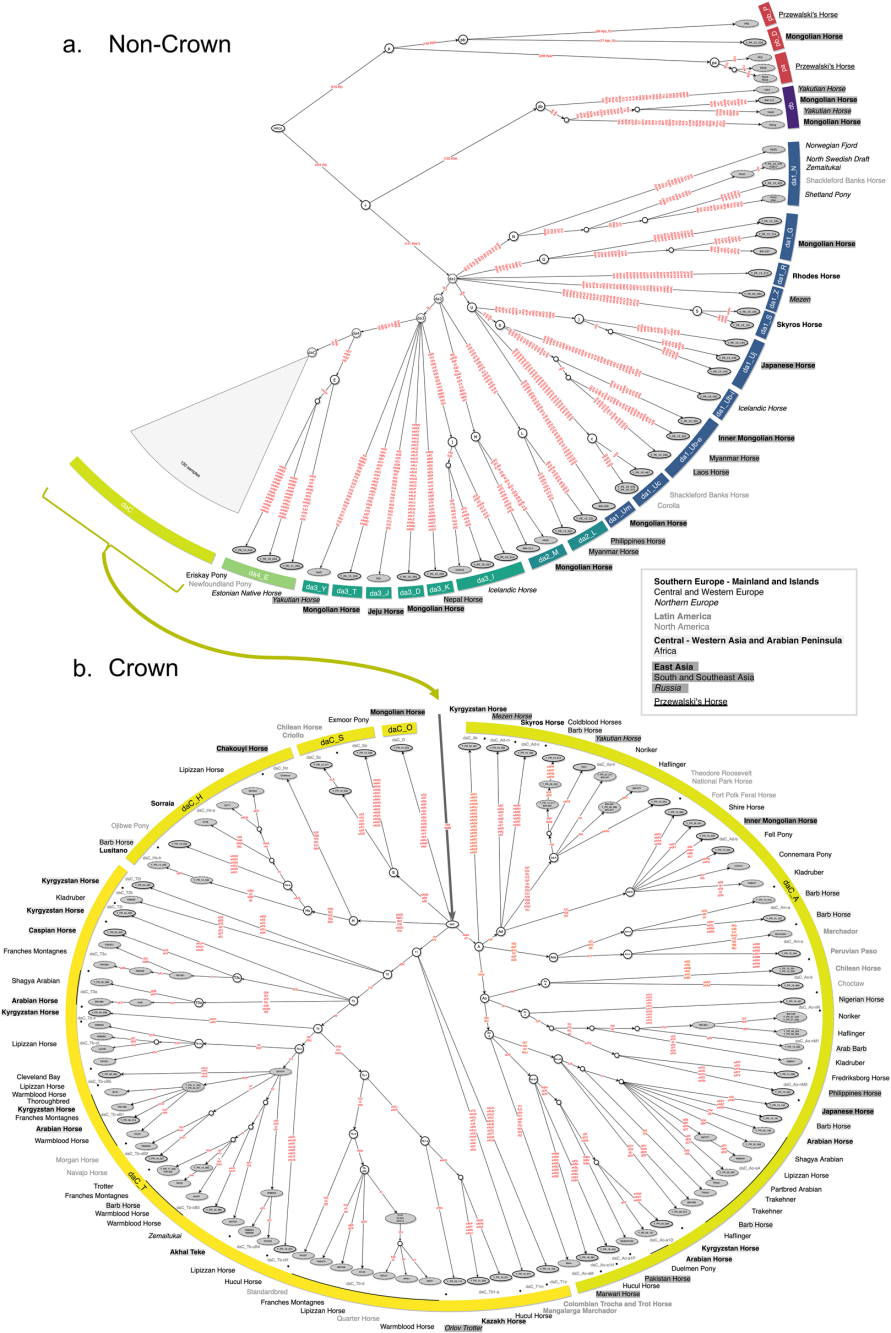
Horse MSY HT topology ‘horseYtree.vs1’. Fine-scaled HT tree from 165 domestic and five Przewalski’s horses tree based on 2966 MSY variants. Variants (‘identifiers’) are given on branches in red. The samples (IDs) are at the tips, and samples carrying HTs first detected in this study are circled with solid lines. mjHGs and breeds are denoted in the outer circle. Breeds are identified by geographic region as indicated in the box. (**a**) HTs outside the Crown. For long branches the number of identifiers is given in parenthesis (full information in Supplementary Table S4). (**b**) Crown HTs. In the Crown, the 39 subhaplogroups (sHGs) are separated with black dots and denoted in grey letters. The black lines in the outer circle mark the previously defined Thoroughbred (dac_Tb-d, dac_Tb-oB3b, dac_T3a, dac_Tb-oB1) and Arabian signatures (dac_Ao-aA, daC_Ao-aA1D)^23,24^. Details on samples, variants and HGs are given in Supplementary Tables S3, S4. The figure is fully readable only in the digital version.

Despite the identification of many new HTs, the sequence diversity on the horse domestic MSY was found to be extremely limited (Table 1). This was especially so when considering intensively managed breeds from Central Europe and the Americas, which mostly carry Crown HTs (Watterson’s θ = 4.57–8.87 × 10^–6^). The lack of genetic diversity precluded further resolution of the phylogenetic relationship among Crown HTs on the basis of SNP variation only. In order to further improve phylogenetic resolution, short insertion and deletion polymorphisms (indels) and a previously described short tandem repeat (STR)^23,24,31^ were included as well as imputed genotypes (see Supplementary Information S1) resulting in a total of 2,966 MSY variants (2781 SNPs, 184 indels, 1 STR). This provided the basis for the refined parsimony tree, ‘horseYtree.vs1’, and the placement of diagnostic positions (‘identifiers’) underlying each individual branch (Fig. 3). Our strategy confirmed 2031 identifiers previously described (r-, s-, f- and q-variants) but uncovered 935 (880 SNPs, 55 indels), that were ascertained for the first time (e-variants; Supplementary Table S4). Overall, the horseYtree.vs1 phylogeny included five HTs in p, four in db and 144 in da (Fig. 3). In the Crown we distinguished 107 HTs from 487 variants in 120 samples, out of which 40 were previously unknown (Fig. 3b). Besides the two new Crown mjHGs (daC_O and daC_S), we detected several former unresolved^24,31^ early branching Crown subhaplogroups (for example daC_Ak, daC_Ad-m, daC_Ad-s or daC_T2r). This demonstrated the wide distribution of Oriental lineages in addition to the previously identified Thoroughbred^22^ and Arabian^23^ signatures.

**Table 1:** Horse MSY HT diversity across geographic regions based on stringently filtered SNPs (related to Fig. 2).

Regions	Samples	mjHGs	Unique HTs	Mean number of pairwise differences	Watterson’s θ
East Asia	20	13	20	187.13 (± 83.66)	8.42 × 10^–5^
East Asia wo* Pb_D	19	12	19	87.57 (± 39.42)	3.17 × 10^–5^
East Asia wo Pb_D/wo db	17	16	17	44.05 (± 20.11)	1.73 × 10^–5^
South and Southeast Asia	8	4	8	45.75 (± 22.25)	1.09 × 10^–5^
Russia	7	5	7	134.28 (± 65.84)	2.60 × 10^–5^
Russia wo db	5	4	5	34.20 (± 18.06)	7.58 × 10^–6^
Northern Europe	14	6	11	42.02 (± 19.42)	1.17 × 10^–5^
Southern Europe Mainland and Islands	6	4	6	34.46 (± 17.57)	6.92 × 10^–6^
North America	13	6	12	34.76 (± 16.21)	8.97 × 10^–6^
Central and Western Europe	66	5	55	17.06 (± 7.68)	8.30 × 10^–6^
Central and Western Asia Arabian Peninsula	14	2	14	17.15 (± 8.12)	5.15 × 10^–6^
Latin America	8	3	7	19.92 (± 9.88)	4.57 × 10^–6^
North Africa and Nigeria	9	3	9	23.44 (± 11.40)	6.47 × 10^–6^
Total	165	26	153		

*wo* without.

### Ancient DNA insights into the emergence of MSY haplogroups

**Figure 4:**
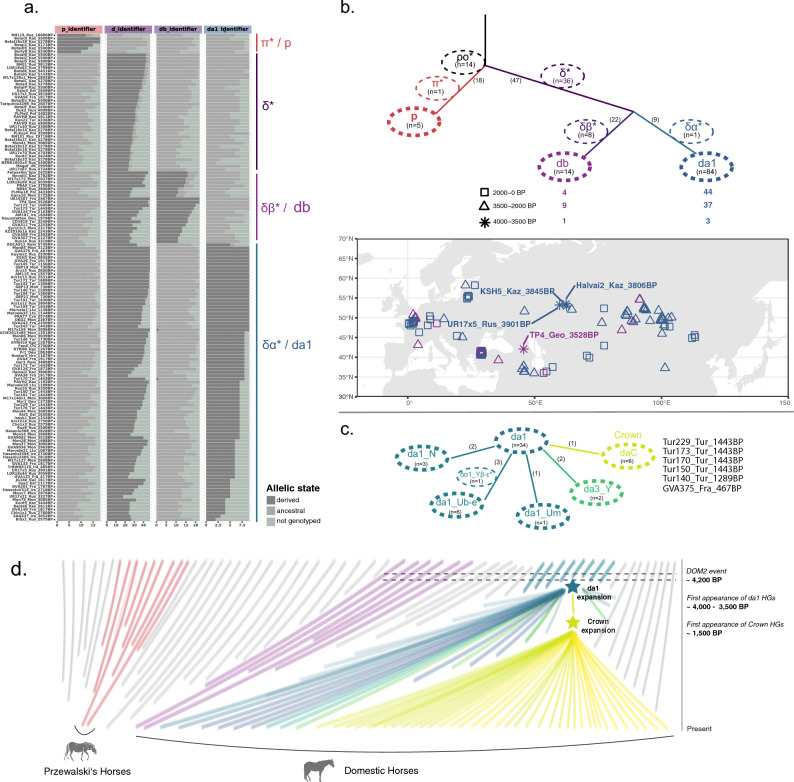
Clustering of ancient samples and emergence of haplogroups. (**a**) Results from grouping of 149 samples (Y-axis) into four clusters based on number of derived/ancestral states of identifier variants (X-axis). (**b**) Illustrated summary of the clustering of all ancient samples (according to panel a). The number of identifier SNPs is given on the branches, and the number of samples in each cluster is given in the dashed circles. Groupings that were only detected in ancient samples are given in Greek letters. The spatiotemporal distribution of the 98 samples clustered in db and da1 is shown in the lower panel. The oldest samples in da1 and db, which are dated to the 4000–3500 BP period, are outlined. (**c**) Clustering of 53 sufficiently sequenced da1 samples into one of the mjHGs. The six samples that clustered to the basal node of the Crown (daC) are given on the right. Full information is given in Supplementary Table S5. (**d**) Hypothesized emergence and spread of Y haplotypes in domestic and Przewalksi’s horses on the basis of currently available data. Coloured lines represent detected, grey lines uncovered haplotypes.

Our refined MSY phylogeny showed that the da-expansion was wider than previously suggested (designated as ‘Dom-West’ in^23^), and that many db branches might have formed in a similar timeframe (Fig. 2a). It has recently been shown that the modern domestic horse (DOM2) was domesticated in the Western Eurasian steppe around 4200 years before present (BP) and does not descend from previous domestic bloodlines emerging at Botai around 5500 years BP^18,20,32^. However, in previous work, estimates of the basal branching point of da (da1) applying the MSY molecular clock^23^, returned a significantly younger timeframe (2600 ± 900 years BP) than for the DOM2 domestication. Radiocarbon date and/or archaeological context associated with ancient specimens^18,20,32^ allowed us to date the emergence of some of the main branches of the MSY phylogeny, without relying on previous molecular clock calibrations (Fig. 4a,b). The majority of the 163 ancient samples showing sufficient coverage clustered into da1 or db (N = 98), and included samples dating back from the 2^nd^ millennium BCE onwards. The three oldest samples clustering within da1 (KSH5_Kaz_3845BP, Halvai2_Kaz_3806BP, UR17 × 5_Rus_3901BP) suggest that this HG already segregated in Russia/Kazakhstan between 4000 and 3500 years BP. In Europe, da1 HGs were also retrieved from a single Bronze age sample (Gar3_Rom_3489 from Romania) and from many sites dated to the last 2000 years (Fig. 4b). Sample TP4_Geo_3528BP proves the occurrence of the db HG in West Asia (Georgia) around ~ 3500 years BP. The ancient samples in db (N = 14) suggest that this clade, which is restricted today to East Asia and Russia, had a broader distribution in early domestic horses until the Sassanid and Byzantine period. Since none of the samples carrying da1 and db were associated with autosomal ancestry profiles characteristic of other populations than the DOM2 lineage^18,20,32^, the MSY DNA evidence analyzed here demonstrates the tight association of the da1 and db HGs with the DOM2 domestication and further spread across Eurasia. It also revealed that the two HGs were formed prior to what was estimated on the basis of molecular clocks (i.e. for da at least ~ 3900 years ago vs ~ 2600 years in^23^).

Outside da1 and db, we clustered 65 samples (Supplementary Fig. S3). Twenty of these, representing a timespan from 36800 to 4000 years BP and a Tarpan sample from the early twentieth century CE obtained from natural history museum collections, have been placed either in p (Botai, ~ 5500 BP; N = 5), π* (Pershinskaya, Upper Paleolithic; RN115_Rus_16686BP) and ρο* (Upper Paleolithic to Bronze Age and the twentieth century Tarpan sample; N = 14). The remaining 45 samples were placed along the Þ*, δα* and δβ* branches of the MSY phylogeny and were mainly dated to the 6000–2000 years BP time range. It is noteworthy that 20 of those samples showed a typical DOM2 autosomal ancestry profile (Supplementary Table S5), which suggests that their observed MSY pattern captures the evolutionary events associated with the domestication of wild horses into the DOM2 lineage. Importantly, the considerable proportion of ancient samples clustering outside da and db confirmed previous work reporting far more MSY diversity within ancient than modern domestic horses^19^. This suggests that the spread and management of DOM2 horses in the past 4000 years was associated with a major change in the Y-chromosomal gene pool (Fig. 4d), leading to the prevalence of the da and db lineages^17,19,33^.

In previous work, we postulated that the rise and spread of the Crown HG were associated with the expansion of Oriental bloodlines^26^, and the most recent common ancestor of the Crown was dated around 1500 ± 500 years BP assuming a molecular clock of 1.69 × 10^−9^ mutations/site/year^23^. To test for the validity of this model, we identified 53 sufficiently sequenced ancient da1 HG samples (Supplementary Information File S1) and checked whether they clustered into any of the mjHGs. A total of 34 such samples showed a basal placement within da1, while the remaining 19 belonged to da mjHGs (Fig. 4c, Supplementary Fig. S4, Supplementary Table S5), including six daC samples, representing the most basal node of the Crown. The oldest such samples originated from the Byzantine site of Yenikapi, Turkey, which covers a time period between the fourth and the eleventh century CE. For one specimen (Tur140_Tur_1289BP) the age has been confirmed to the seventh/eighth century CE by radiocarbon dating^20^. Therefore, ancient DNA evidence is congruent with previous dating estimates for the emergence of the Crown group.

### Unique Y diversity in indigenous horse populations

Our refined MSY tree revealed the presence of several unique Y lineages within indigenous populations. Some such mjHGs were supported by at least two samples from a given region (for example, da1_G and da1_Uj in in Mongolia and Japan, respectively), while others were detected across a wider geographic range (for example, da1_Ub-e in Myanmar, Laos and Inner Mongolia) (Fig. 3, Supplementary Table S3). Moreover, the phylogenetic placement of a cryptic HT previously detected in Estonian Native horses^29^ is now resolved to define one new mjHG (da4_E) together with the Eriskay and the Newfoundland Pony. Also, former hidden private Y variation in Japanese horses^30^ and inner Mongolian horses^28^ and several other regions were ascertained (Fig. 3). Some indigenous horses grouped nested within modern breeds (for example the Inner Mongolian horse in daC_Ad-b, the Japanese Horse in daC_Ao-aA) and as ‘horseYtree.vs1’ delineates the chronologic descent of the lineages, this pattern is in coherence with a recent cosmopolitan descent of those lineages.

Overall, Mongolian and Inner Mongolian horses were found to exhibit the most diverse range of MSY HTs, with 14 HTs distributed over 10 mjHGs (Supplementary Table S3). This is not only in line with the pronounced genetic diversity detected in Mongolian and Chinese horse populations reported in previous studies^34–37^, but indicates that horse management in these regions preserved some of the original diversity of paternal lineages and was not characterized by the intensive selection of specific stallion bloodlines. Interestingly, one Mongolian horse carried an MSY HT (pb_D) clustering with Przewalski’s horses’ HG (Fig. 2). All todays Przewalski’s horses descend from twelve wild-caught individuals and in a recent study, representing all founding lineages, only two well separated Y-HTs were detected^38^, which were both represented in our dataset (pa and pb_P). Based on the HT topology, showing clear distinction of pb_D from the HTs segregating in the current Przewalski’s horse population (Fig. 3a), the pb_D individual is unlikely the result of a recent hybridization between a Przewalski’s stallion and a domestic mare. The finding instead suggests past introgression of an HT that is not detectable in the current population of Przewalski’s horses anymore or provides evidence for the previously-reported leaky genetic isolation post-divergence^38^.

It is also noteworthy that, outside of Asia and northern Europe, non-Crown mjHGs were only detected in samples from populations inhabiting islands (Greece—Skyros, Rhodes; North America—Shackleford Banks/Corolla). In the context of Skyros Horses, which carried three private HTs, the Y signature is in congruence with autosomal data, where Skyros horses remained isolated without any close relationships with modern breeds or Middle Eastern populations^39^. The MSY data underline that the Skyros as well as Rhodes horse populations have been spared to some extent from recent breeding influences.

### The spread of mjHG da1_N

**Figure 5:**
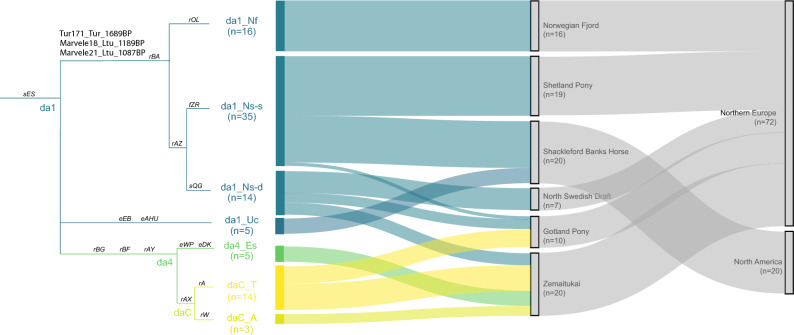
MSY lineage tracing in breeds carrying mjHG da1_N. On the left the illustrated tree based on 16 identifiers discriminating the HGs detected in six breeds carrying mjHG da1_N is given, with identifier variants on branches. The three ancient samples that clustered into the da1_N are denoted on its basal branch. On the right, the frequencies of each haplogroup in the studied breeds and their geographical region are shown in a Sankey diagram.

Our new MSY phylogeny not only reveals the true extent of the MSY diversity in modern and ancient horses but also allows us to track the geographic spread of specific haplogroups after horse domestication. The mjHG da1_N provides a clear example about how this approach can provide such new insights. As this mjHG was first detected in Shetland Ponies and Norwegian Fjord Horses^26^, and the North Swedish draft^23,25^, it was hypothesized to reflect ‘Northern European’ origins. Our initial screening data confirmed the presence of da1_N in five breeds from Northern Europe but also identified it in horses from the Baltic region and the Shackleford Banks Horse from North Carolina (USA) (Fig. 1, Supplementary Table S2). We genotyped a collection of 92 males, representing six da1_N harbouring breeds, for 16 HG identifiers. We could further split da1_N carriers into three HGs (da1_Nf, da1_Ns-s and da1_Ns-d) and re-assigned 27 individuals to four other mjHGs (Fig. 5; Supplementary Table S1, Supplementary Table S2). The da1_Nf HG was found to be private to Norwegian Fjord (in congruence with breeding history^22^), while da1_Ns-s was detected in Shetland Ponies, Gotland Ponies and horses from Shackleford Banks. Such phylogenetic clustering indicates that at least a fraction of the horses from Shackleford Banks trace their origins into da1_Ns ancestors from Northern Europe, that were likely imported to the region as part of the colonization history of the continent^40,41^. All North Swedish drafts were found to carry the da1_Ns-d HG, which was also detected in Zemaitukai horses and a few Gotland ponies. Allover, MSY pattern harmonize with breeding strategy: the isolated breeding in Norwegian Fjords, Shetland Ponies and North Swedish draft, was reflected in the single HG detected. This contrasted to the mixed ancestry in the Gotland Pony and in the Zemaitukai breed. The latter showed four mjHGs most of which (da1_Ns-d as well as the Crown mjHGs daC_A and daC_T) can be explained by recent breeding influences. In addition, we detected da4_Es in the Zemaitukai, showing a common ancestry with the Estonian Native Horse. We did not detect any recently introgressed HTs in Shakleford Banks horses, as we found only two, rather unique, HGs: the above described da1_Ns-s and da1_Uc, a HG shared with Corolla horses, another feral population on Outer Banks. In the ancient dataset we detected da1_N via identifier ‘rBA’ in two samples from the Marvele medieval cemetery in Lithuania (Marvele21_Ltu_1087BP, Marvele18_Ltu_1189BP), dated to the eighth to the eleventh century CE, and a sample from Yenikapi (in present-day Istanbul; radiocarbon dated to 260–395 CE) (Supplementary Fig. S4).

While da1_N was not surprising in the medieval Marvele samples, the Yenikapi finding was rather unexpected. But this observation is an explicit example of how dynamically horses were moved after domestication. As shown here, refined MSY HT tracking based on contemporary breeds is very powerful in revealing recent stallion-mediated population dynamics, whereas a comprehensive collection of ancient samples will be required to elucidate the distribution and impact of stallions in early periods.

## Concluding remarks

The refined MSY phylogeny reconstructed in this study improves our current understanding of the horse domestication process and provides a robust scaffold for further investigations. The careful selection of the individuals sequenced helped us to circumvent the ascertainment bias that impacted previous reconstructions, thereby offering a more complete characterization of the diversity of paternal lineages present both in ancient and modern, as well as global and local breeds. That the vast majority of the MSY HTs identified mainly descend from DOM2 ancestors is in line with current understanding of the domestication process and provides resolution into tracking the spread of influential individual lineage through space and time, especially within the recently established Crown group. The possible addition of faster evolving markers, such as STRs, may further enhance resolution and enable individual ancestry tracing^42,43^. Our work unveiled severe drops of MSY genetic diversity during horse domestication and selective breeding but also revealed modern populations that retain unique Y HT diversity, especially in Asian and insular populations. Future work will uncover the distribution of the defined HGs and reveal the extent to which other lineages remain to be characterized. MSY haplotyping may prove an important decision-making tool for breed management and for defining conservation priorities.

## Material and methods

A detailed description of methods including program parameters and program versions used is available in the Supplementary Information File S1.

### Ethics statement

The study was discussed and approved by the institutional ethics and welfare committee of the University of Veterinary Medicine Vienna in compliance with GSP guidelines and national legislation (ETK-10/05/2016). The research was performed in accordance with relevant guidelines reported in the above-mentioned document. Samples were taken strictly respecting the animal welfare standards in the respective countries and consisted of (i) hair roots, (ii) retained material from paternity testing (iii) or archival blood collected > 20 years ago. Biosample responsibilities of authors are in Supplementary Table S2 and Supplementary Table S3. All the samples of this project are coded. The study was carried out in accordance with ARRIVE guidelines.

### Distribution of MSY HGs/HTs in modern samples

#### Sample sets

For checking the distribution of the major HGs (mjHGs), we designed a dataset composed of 1537 male horses representing 135 domestic horse breeds/populations and the endangered Przewalski’s horses. With this selection, we aimed to comprehensively represent the geographical and phenotypic distribution range of domestic horses. In order to cover as many male lineages per breed, pedigree tail male line information was considered in the sampling design, when available. A maximum of 15 samples were taken per breed/population, while more samples were enclosed from East Asian populations due to a lack of pedigree information and expected population substructure. We included samples that were already used in previous Y studies^23,24,27–29,31,44^ and in addition, we added samples collected for the purpose of diversity analysis in several different projects. If applicable, we selected samples from separate sample collections, with regard to region, timepoint and research groups. We also accessed samples of animals from previous generations, which were stored in archives in the form of frozen blood vials. The full information of the genotyping dataset is given in Supplementary Table S2. Samples were classified into 10 different geographical regions based on breed origins or sampling location. For fine-scaled genotyping, we further sampled six breeds each showing some members carrying a da1_N HT (in total 92 samples).

#### Genotyping

Distribution of HGs/HTs was inferred by genotyping a selection of MSY markers defining major nodes of the MSY phylogeny. Specifically, we considered a total of 16 markers identified from^23^ for defining the 10 mjHGs used for those analyses focused on the worldwide distribution, and a total of 16 other markers defining those HTs most frequent in Northern Europe. Information on selected markers and backbone HT/HG structure is provided in Supplementary Table S1. Competitive allele-specific PCR (KASP™, lgcgroup.com) was used for genotyping a total of 1537 and 92 horses in both analyses, respectively, and allelic states at genotyped markers were concatenated to form individual HTs. The distribution of haplotypes was visualized with R version3.6.1. KASP™ screening was also used for validation of Y specificity and allelic state for 256 SNPs and short indels (marked in Supplementary Table S4).

### Constructing MSY haplotype topologies

#### Whole genome sequencing data

A total of 55 males were selected from^23^ to represent all previously known horse MSY HTs. The ancestral states of variants was identified using publicly available data from a WGS male donkey^45^ (Supplementary Table S3).

#### Target enriched sequencing data

We performed target enriched sequencing (TES) of 5.063 Mb single-copy Y (scY) regions (Supplementary Table S6), as described in Supplementary Information File S1. A total of 76 male samples were selected based on the KASP genotyping results, their geographic location and/or breed and pedigree information (Supplementary Table S3). The data obtained for the 76 samples characterized in this study were supplemented with TES data from 39 specimens previously analyzed using the same experimental procedures^24^.

### Data analysis

#### Mapping and ascertainment

TES and WGS NGS data were mapped to the LipY764 assembly (GCA_002166905.2) using bwa aln^46^ and variant calling for was performed with freebayes^47^, and further filtered using customized scripts (Supplementary Information File S1 and Supplementary Fig. S1).

#### Genotyping NGS data

The 2081 SNPs and 113 indels defined in TES dataset, and the 1891 SNPs and 103 indels identified in the WGS dataset were united with those MSY variants previously reported (2094 SNPS and 172 indels, respectively)^23,24^. The resulting 3009 SNPs and 232 indels were genotyped separately in the horse NGS dataset (N = 170) and in the donkey with freebayes^47^, and filtered (described in Supplementary Information File S1). Details including coordinates, reference and alternate allelic states, variant IDs and the final genotypes characterized for the 3149 polymorphic positions (representing 2940 SNPs, 208 indels, and 1 STR) are given in Supplementary Table S4.

### Haplotype trees

A robust parsimony tree was constructed based on 2678 stringently filtered SNPs (Supplementary information file S1 and Supplementary Table S4). In order to reach maximum resolution, we included also short indels and imputed missing positions (see Supplementary information file S1 and Supplementary Fig. S2), leaving a total of 2966 variants (2781 SNPs, 184 indels, 1 STR) for reconstructing the refined ‘horseYtree.vs1’ phylogeny. Variant nomenclature from previous studies was retained (ie. first description of r-^26^, s-^25^, f-^23^ and q-^24^ variants) while those variants defined here the first time were marked with a ‘e-’ prefix (Supplementary Table S4). Those variants determining HT at specific branches of the horseYtree.vs1 (hereafter referred to as ‘identifiers’) are described in Supplementary Information File S1.

### Analysis of ancient samples

#### Sample set

Ancient horse NGS data mapped to MSY contigs were obtained from 3 different studies Gaunitz et al.^18^ (PRJEB22390), Fages et al.^20^ (PRJEB31613) and Librado et al.^32^ (PRJEB44430). In total, BAM alignment files of 282 male horses were downloaded. Metadata associated with each ancient specimen is provided in Supplementary Table S5. A fraction of the downloaded sequence alignments was mapped against the 1.46 Mb reference ‘chrY’ (N = 134)^18,20,26^, while the remaining fraction (N = 148) was mapped against the LipY764 assembly^23^. Details are given in Supplementary Information File S1.

#### Placing ancient samples on the modern phylogeny

To place ancient samples into the modern MSY phylogeny, we first considered a total of 1031 ‘identifier variants’ diagnostic for clusters p, d, db or da1 (schematically shown in Fig. 4b). Presence/absence, position and orientation of the identifier variants on the ‘chrY’ were determined by mapping their flanking regions to the chrY reference. This led to the identification of 232 variants that could be defined on both references. We next used the sequence alignment to genotype the 282 ancient samples using GATK HaplotypeCaller^48^ version 4.1.4.1. Due to the uneven coverage achieved across all ancient samples, only a total of 96 identifier variants could be genotyped on both references, leaving a total of 169 samples that could be genotyped for at least 48 (50%) identifier variants. Those samples were clustered based on the number of derived state counts for the respective identifiers on branches (18 p-, 47 d-, 22 db-, and 9 da1-identifiers). If a sample revealed at least half of the variants in derived states and no ancestral states for a branch, the sample was assigned to the underlying group identified amongst present-day horses. Samples showing both, ancestral and derived states for variants on a branch, were clustered to their own internal branching points represented by ancient samples only (denoted by Greek letters and ‘*’). Ancient samples clustering in da1 were further checked for additional identifiers defining mjHGs in da1 (Fig. 4c). Details about clustering of ancient samples can be found in Supplementary Information File S1.

## Supplementary Information

Supplementary Information 1.

Supplementary Information 2.

Supplementary Information 3.

Supplementary Information 4.

Supplementary Information 5.

Supplementary Information 6.

Supplementary Information 7.

## Data Availability

Mapped reads (to lipY764) of samples sequenced in this project have been submitted to ENA archive project id PRJEB55914 (https://www.ebi.ac.uk/ena/browser/view/PRJEB55914). Mapping data for previously published samples and the chrY reference in fasta format and are reachable on Zenodo (https://zenodo.org/record/7704382#.ZAn-COzMJQP; https://doi.org/10.5281/zenodo.7704382).
