# Supplementary material for: Refining the evolutionary tree of the horse Y chromosome
Source: Sci Rep. 2023 Jun 2;13:8954. doi: 10.1038/s41598-023-35539-0 (PMC10238413; doi:10.1038/s41598-023-35539-0)
Supplement: Supplementary file 1 — Supplementary Information 1. [file 41598_2023_35539_MOESM1_ESM.docx]

**Refining the evolutionary tree of the horse Y chromosome**

Elif Bozlak, Lara Radovic, Viktoria Remer, Doris Rigler, Lucy Allen, Gottfried Brem, Gabrielle Stalder, Caitlin Castaneda, Gus Cothran, Terje Raudsepp, Yu Okuda, Kyaw Kyaw Moe, Hla Hla Moe, Bounthavone Kounnavongsa, Soukanh Keonouchanh, Nguyen Huu Van, Van Hai Vu, Manoj Kumar Shah, Masahide Nishibori, Polat Kazymbet, Meirat Bakhtin, Asankadyr Zhunushov, Ripon Chandra Paul, Bumbein Dashnyam, Ken Nozawa, Saria Almarzook, Gudrun A. Brockmann, Monika Reissmann, Douglas F. Antczak, Donald C. Miller, Raheleh Sadeghi, Ines von Butler-Wemken, Nikos Kostaras, Haige Han, Dugarjaviin Manglai, Abdugani Abdurasulov, Boldbaatar Sukhbaatar, Katarzyna Ropka-Molik, Monika Stefaniuk-Szmukier, Maria Susana Lopes, Artur da Câmara Machado, Valery V. Kalashnikov, Liliya Kalinkova, Alexander M. Zaitev, Miguel Novoa‐Bravo, Gabriella Lindgren, Samantha Brooks, Laura Patterson Rosa, Ludovic Orlando, Rytis Juras, Tetsuo Kunieda, Barbara Wallner

The Supplementary Information File contains

1. Legends to Supplementary Tables
2. Four Supplementary Figures
3. A detailed description of the methods

# 1. Legends to Supplementary Tables

## Supplementary Table 1

Gives detailed information on 24 HT identifiers used in worldwide (16) and/or da1_N clade (16) genotyping. For each marker ID, coordinates on LipY764, Ref/Alt Allele, the ancestral state, polymorphism type and the HG the identifier determines are given. The allelic combinations for the revealed and the ancestral (root) HTs are given for both genotyping setups in the lower panel.

## Supplementary Table 2

This table lists breeds/populations used for genotyping. Sheet 1 provides the underlying data for the worldwide genotyping shown in Figure 1. Number of samples from each geographical region and results are given in columns B-Q. The breeds/populations analyzed with number of samples included and sample collection responsibilities are given in column R. Sheet 2 gives number of samples and genotyping results in the da1_N population genotyping shown in Figure 5. Sample collection responsibilities are listed in column K.

## Supplementary Table 3

A full list of 171 NGS samples: key ID; breed/population; geographical region of origin; remarks on type; paternal lineage information if available; responsibilities for sample collection; study where the sample was used first; MSY HT defined in previous study; mjHG in Figure 1; HT in horseYtree.vs1; raw data source (whole genome sequenced (WGS) or target enriched sequenced (TES); Biosample ID; average mapping coverage on LipY764 scY regions.

**Supplementary Table 4**

Variant table. Sheet 1 summarizes information for the 3,149 good quality Y-chromosomal variants used in this manuscript. The table is hierarchically clustered, meaning samples in the same HG are given next to each other, and variants are also ordered in lanes based on this clustering.

The information is divided into five sections: 1) General variant information on variants in columns B-G (grey): coordinates, variant ID, REF/ALT allele, variant type (SNP/indel/STR), first study variant was described. 2) Genotyping results (orange) in columns H-FW: allelic state in 170 horses (for those samples rawdata, breed and HT are provided in the header), the donkey (DON) and the reconstructed ancestral state. ‘0’ denotes REF Allele, ‘1’ denotes the ALT Allele, ‘.’ not called. Derived alleles are shaded in grey. 3) Information relevant for generating the parsimony tree and the imputation for generating horseYtree.vs1 (Column FX-GF, yellow). The position of variants on branches in horseYtree.vs1 is given in column GF - ‘identifier for Hg/HT’. 4) Information for lab validation and genotyping (columns GG-GJ, blue). 5) Information for ancient analysis (columns GK-GN, green) including selected identifiers, position and orientation on ‘chrY’ reference.

Sheet 2 gives 72 bad-quality variants which were discarded from the analysis.

Sheet 3 is the imputed input file for horseYtree.vs1 (2,966 variants) in binary format (‘0’ REF, ‘1’ ALT allele).

**Supplementary Table 5**

Ancient data analysis table. This data contains raw data source and metadata for the 282 ancient samples, allelic states at identifier variants (ancestral, derived, not called) and the clustering. The metadata for 282 male horse samples were assembled from the original articles: Gaunitz et al., 2018, Fages et al., 2019, and Librado et al., 2021. Sample ID is given in column A-B (grey), raw data sources are given in columns C-G (green), metainformation on samples in columns H-O (blue), Y-chromosomal clustering in columns P-R (yellow); genotyping results for the 105 identifier positions are given in columns T-DT (color code according to Figure 1/2/3).

**Supplementary Table 6**

Information on bait regions for target enrichment on LipY764 used. LipY764 contig_ID, bait start, bait end and bait length is given.

# 2. Supplementary Figures

**Supplementary Figure S1**

**Workflow for variant ascertainment, NGS genotyping and constructing phylogenetic trees.** The number of variants was given after each analysis/filtering step. The variant ascertainment was performed separately on TES and WGS datasets. For genotyping, variants from previous studies were merged with the newly ascertained ones and SNPs (red) and indels (green) were analyzed separately.

**Supplementary Figure S2**

**Workflow for imputation from hierarchical clustering**. The original variant file contains missing positions. Based on the robust MP tree (1), samples were grouped with the information from the tree (2). Due to complete linkage on the MSY, the order of the variants can be changed. We used this info to generate a hierarchically clustered file including samples and variants. This file shows straightforwardly which samples are clustering together and which variants bring them together (3). We then imputed the missing positions on inner branches if the clustering of the sample was supported by (a) subsequent variant(s) located on terminal branches of the tree (4). Variants that were not possible to impute straightforwardly were discarded.

**Supplementary Figure S3**

**Ancient samples clustering not in da/db.** Full information on samples is given in Supplementary Table 5. a) Geographic and temporal distribution of the 20 ancient samples clustering in ρο* (n=14), π* (n=1), and p (n=5). Sample names were given for all samples and those include sample id, sampling country (present), and the years before present (BP). Different colors denoted haplogroups. b) 45 Ancient samples clustering in δ* (n=36), δβ* (n=8) and δα* (n=1). Samples from different periods were given in different shapes. Two timeframes (6,000 – 4,000 BP and 4,000 – 2,000 BP) and two outlier samples (1,917 BP & 19,719 BP) are given. Colors denote the clusters.

**Supplementary Figure S4**

**Clustering da1 samples into mjHGs**

Genotyping results in the 53 samples that were analyzed for clustering into da1 mjHGs. Identifier SNPs are given on the X- and the samples on Y-axis. Variant states are presented in different colors, and the numbers refer to the number of reads supporting the pattern. Final clustering is given on the right. The six samples that carried only one read support for clustering into a mjHG (shown with dashed lines) were clustered conservatively in da1 basal.


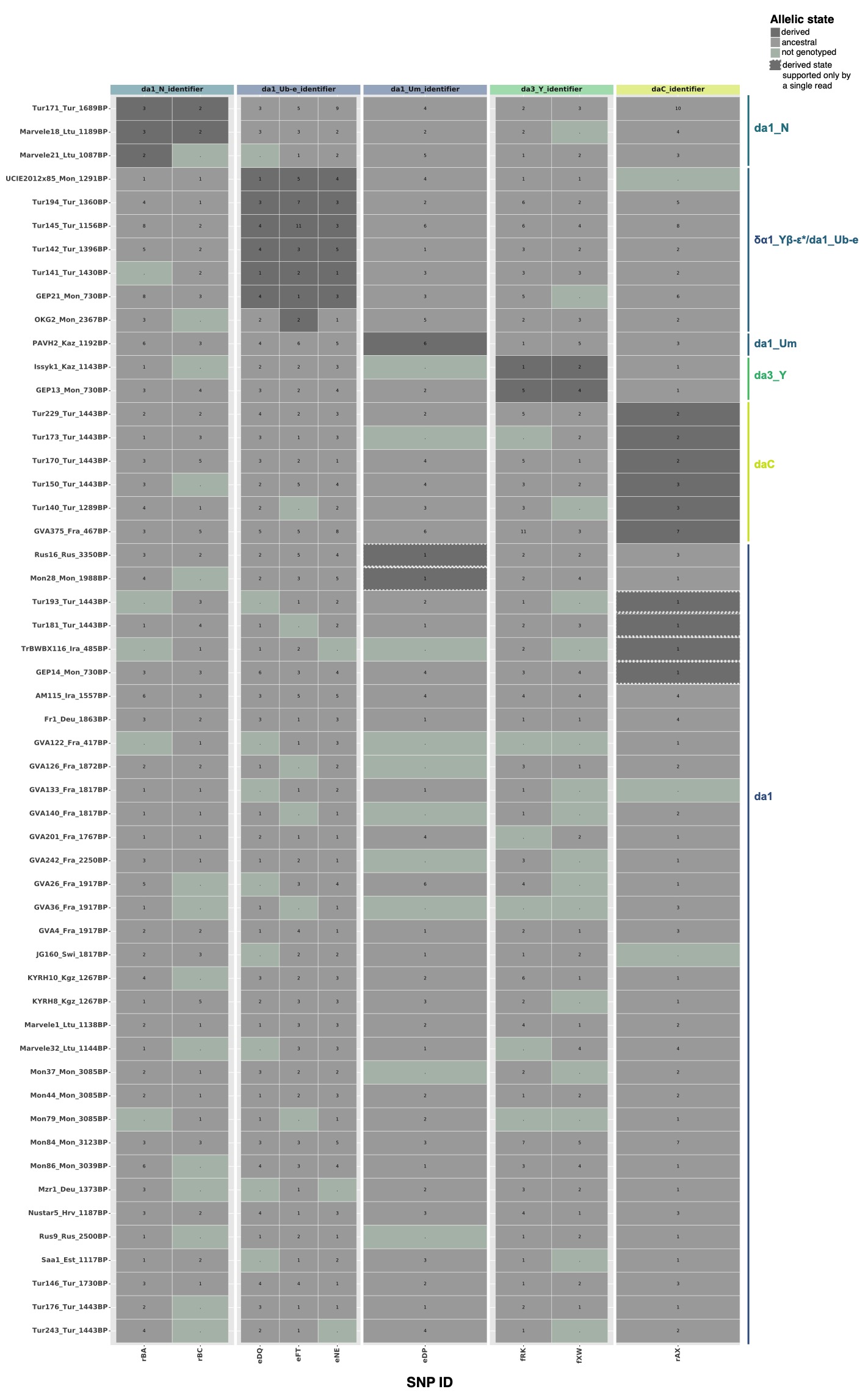


# 3. Detailed description of methods

## Adjusted Haplogroup and Haplotype Nomenclature

We needed to adjust previously established HG nomenclature, in order to disclose the clustering of HTs/HGs unequivocally in the naming system. We therefore labelled the cluster that contains most domestic horses (former stated as DomWest [1]) as ‘da’. Sequential branching points in ‘da’ were consecutively numbered (da1-da4), and we termed the Crown HG as ‘daC’. The previously described distantly grouping HG O [1–3] was shifted to ‘db’. After the first two or three letters, followed by an ‘_’, we tried to keep and extend the ‘four letter code’ established through several previous articles. This four-letter code is deliberately not in alphabetical order but rather informs in which breeds/populations the HG/HT was first described. Accordingly, previous Northern European HG ‘N’ is now termed as ‘da1_N’, the crown HT Ad-hA is now ‘daC_Ad-hA’. In the refined tree, we introduced internal numbers into the previously established nomenclature system in case we defined new inner branching points. This was conducted to keep the previous nomenclature visible: for example, ‘Ta-bA’ is now ‘daC_T3a-bA’. For further sub-branches, we used a successive letter/number system according to human guidelines for MSY HTs [4] (like daC_Ao-aA1a2a).

| Examples | Previous HT nomenclature | New HT Nomenclature |
| --- | --- | --- |
| Przewalski’s Horse HT | Pz-b | pa_A |
| Mongolian Horse | Oa-a | db_Ma |
| Yakutian Horse | Oa-b | db_Mb-y |
| Norwegian Fjord | Nf | da1_Nf |
| Mongolian Horse | M | da2_M |
| Jeju Horse | J | da3_J |
| Thoroughbred | Tb-dW1 | daC_Tb-dW1 |
| Noriker | Ad-hA1 | daC_Ad-hA1 |
| Barb | Hs-b | daC_Hs-b |

## KASP genotyping

KASP™ screening was performed as described by the supplier (LGC, Berlin Germany) on a CFX96 Touch™ Real-Time PCR Detection System. Genomic DNA was isolated with nexttec® (Hilgertshausen, Germany) and used with a concentration of 3 ng/µl. Each run included samples with their allelic state known as positive controls, while DNA from females and non-template controls were used as negative controls. Raw data were analyzed with Bio-Rad CFX Manager 3.1® software (Biorad, Vienna, Austria).

## Target enriched sequencing of MSY regions

Genomic DNA was isolated from the Biosample given in Supplementary Table 3 for each individual using DNeasy Blood and Tissue Kit (Qiagen, Vienna, Austria). For NGS library preparation and target enrichment, the Custom SureSelectXT low input target enrichment from Agilent, Vienna, Austria, was used. As previously described [1] the LipY764 assembly (GCA_002166905.2) harbors 5.8 Mb of so-called single-copy Y (scY) regions that were shown to be suitable for unambiguous variant calling. Baits were generated which covered 5.063 Mb of scY regions of the LipY764 assembly, the mtDNA, and some autosomal loci. The 4,032 Y-chromosomal bait segments that cover 86.7% of the LipY764 scY regions are given in Supplementary Table 6. Indexed libraries were generated, and the enrichment was performed according to the protocol supplied by Agilent. Libraries were pooled and sequenced on two Illumina NextSeq550Medium PE150 runs (San Diego, CA, USA) at Vienna Biocenter Core Facilities, Vienna.

## Constructing Y haplotype topologies

### Trimming and mapping

The TES 115 were demultiplexed and adapters were removed with AdapterRemoval (ver. 2.3.1) [5]. Quality-based trimming was performed with ReadTools (ver. 0.2.1.r_716422a3) [6] in default mode for 115 TES, 55 WGS samples (170 in total) and donkey (details Supplementary Table 3). Quality trimmed reads were mapped to the custom reference consisting of the LipY764 contigs (GCA_002166905.2), the X chromosome and the mtDNA sequence from the EquCab3 (GCA_002863925.1). The X chromosome was included as this reduced noise in the mappings in X degenerate regions of the Y chromosome. Mapping was performed by bwa (ver. 0.7.17) [7] aln function with "-n 0.02 -l 200" parameters. Reads which were unmapped, not properly paired, having mapping quality lower than 20 and the duplicates were removed with samtools (ver. 1.10) [8]. Only reads passing these filters and mapping to the Lip764 assembly were further analyzed.

### Variant calling and filtering

Variant calling was performed with freebayes [9] whereas mapped 115 TES and 55 WGS data were provided as two separate batches to the algorithm. freebayes was run with "-p 1 --genotype-qualities" mode. Due to the complex sequence structure of the Y-chromosome and the low expected sequence variation customized ascertainment and filtering is needed to get satisfying signal to noise ratios. Output .vcf files were stringently filtered as follows: 1) only variants having alternative state in at least one individual were kept from the output files with vcftools (ver.0.1.17) [10]; 2) complex variants and variants showing multiallelic states were removed with bash command and bcftools (ver. 1.12) [11] respectively ; 3) remaining variants were normalized with the bcftools (ver. 1.12) norm function; 4) only variants located in the scY regions defined in and not located < 50 base pairs from contig ends were kept with vcftools (ver.0.1.17) ; 5) based on the distribution of the phred-scaled quality scores in the remaining variants, we decided to only keep the ones with a value > 100 for further analysis with vcftools (ver.0.1.17) ; 6) Variants showing alternative states in all the samples were considered as reference errors and removed with bash command.

After that stage, SNPs and indels were filtered separately in both TES and WGS datasets (‘custom filtering’ in Supplementary Figure S1). With this approach we intended to get rid of noise (mostly false positives) coming from different sources.

First, for each variant the following statistics were calculated: number of samples not genotyped; number of alternative and reference read counts; number of samples carrying the alternative allele; mean read depth and mean genotype quality. Based on the distribution of these values different thresholds were chosen for each scenario (TES/WGS/SNPs/indels). The criteria used for ‘custom filtering’ were the following:

- SNPs and indels defined in 115 TES samples --> variants that were not called in at least 20 samples for SNPs and 30 for indels; having heterozygous state in more than 11 samples; having mean depth value < 7.5 and mean genotype quality < 90 were filtered out.
- SNPs and indels defined in 55 WGS samples --> variants that are not called in at least 6 samples for SNPs and indels; having heterozygous state in more than 3 samples; having mean depth value < 5 and mean genotype quality < 90 were filtered out.

A custom Python3 [12] script was used for filtering. Filtering strategy and number of variants remaining after each filtering step are given in Supplementary Figure S1.

### Genotyping NGS data

The SNPs/indels defined in TES dataset (2,081/113) and WGS dataset (1,891/103) were united with the variants (2,094/172) previously reported in [1,13] (see Supplementary Figure S1 for workflow) and the resulting 3,009 SNPs and 232 indels were genotyped separately in the horse NGS dataset (n=170) and in the donkey with freebayes [9] in ploidy 1 mode. The output files were normalized, and variants showing no alternative calls or carrying multi-alternative states were removed as described above. Genotyping results for SNPs and indels from WGS, TE and donkey were merged (in total 3,221 variants). We removed 72 variants because of unambiguous genotyping results among the 170 horses. Those were recurrent mutations, consecutive variants (located on the same contig with < 20 bp distance and having the same allelic state in samples carrying the same HT), SNPs giving unclear signals for clustering or were ambiguous in IGV [14] visual check. Details on the further analyzed 3,149 variants including coordinates, reference/alternative states, variant IDs and genotyping results are given in Supplementary Table 4 sheet 1 and coordinates of the 72 variants that were not considered further are given in Supplementary Table 4 sheet2.

### Haplotype trees

#### Elementary SNP tree

Only the SNPs were considered for creating a first phylogenetic tree and out of the 2,940 SNPs, 262, which were either not genotyped in > 10 in TES samples or > 5 WGS samples were not included. A fasta alignment file based on the remaining 2,678 SNPs containing the 170 horses was created. MEGAX [15] was used for constructing a Maximum Parsimony tree using the following parameters: MP search method: TBR, number of initial trees: 10, MP search level: 1, maximum number of trees to retain: 100. Final mid-point rooted tree was visualized by FigTree (v.1.4.4) [16].

#### Diversity Analysis

The mean number of pairwise difference within each geographic region were calculated with Arlequin (v.3.5.2) [17] based on the 2,678 SNPs in the robust parsimony tree. Watterson’s Theta was calculated according to [18] using R (ver.3.6.1) [19] based on the 2,678 SNPs detected in 5.063 Mb of scY regions.

#### Generating the refined ‘horseYtree.vs1’

The filtered variant file which including the allelic states of 3,149 variants (2,940 SNPs, 208 indels, 1 STR) in 170 horses and the donkey was used as starting point (Supplementary Table 4).

*Classification*

First, samples were grouped according to their clustering in the robust SNP tree, and variants were ordered based on their information content in clusters. This can be conducted as all MSY variants are assumed to be in complete linkage and thus the order of variants on the chromosome can be neglected. The ancestral/derived state was assigned for each variant: for variants determining horse HT clusters (da/db) the ancestral state was deduced from the Przewalski’s horse, for the Przewalski’s HT clusters (pa/pb) from horses and for the Przewalski’s horse/domestic horse separating variants from the donkey. After the classification, the variants clustering samples together into particular HGs/HTs, which is synonymous to the position of variants on the tree branches, is determined (indicated as ‘Identifier’ for each variant).

*Imputation*

Based on the classification, we then imputed the missing positions in the sequencing data. There were two main reasons for the missing positions: i) scY regions which were not covered in the TES data and ii) low covered regions in the WGS data. As we know the hierarchic order of descent for the MSY variants, missing genotype calls in variants located on internal branches can be unambiguously added when the clustering of the sample is supported by (a) subsequent variant(s) located on terminal branches of the tree. Variants, where imputation was problematic (mostly because of no terminal variants supported the clustering) were not included in the horseYtree.vs1. The reason for not including is given for those variants in Supplementary Table 4 (Column GE). Allele sizes at STR fBVB were determined in TE sequenced samples prior imputation by genotyping in the lab as described in [1,13]. An illustration summarizing classification and imputation strategy can be found in Supplementary Figure S2. Out of the 2,966 variants in 170 horses (in total 504,220 positions), 16,257 positions (3.22 %) were imputed with a mean of 24.43 positions (standard deviation 36.41) in WGS samples and a mean of 129.68 position (standard deviation 18,05) in TES samples.

#### Visualization

For generating the tree, allelic states after imputation were catenated and converted to binary format. A median joining network was drawn with Network (ver. 10) [20] with SNPs, Indels and STR equally weighted. The structure was redrawn with draw.io.

***Ancient data analysis***

***Data set***

The downloaded WG bam files were sliced for the data mapped to Y chromosomal contigs. For the samples which were sequenced in more than one study the latest release (PRJEB44430) was used. New sample names were introduced by merging original sample name, the country of sampling (at present) and the age of sample (Supplementary Table 5 column A). Ancient WGS data were mapped to two different references. Reference ‘chrY’ is a catenation of the 1.46 Mb spanning 2,491 MSY contigs (‘nonrepMSY’, PRJNA353919 in total 1.46 Mb) assembled in [21] (see [22] for details). The reference file ‘chrY.fasta’ is uploaded along with this manuscript. The ‘nonrepMSY’ assembly can be seen as a precursor of LipY764. It was generated from the same Biosample (the Lipizzan stallion Lip113) and it has been proven that the ‘nonrepMSY’ contigs are almost fully represented by LipY764 contigs, but orientation of contigs is not consistent between the two references [1].

***Defining identifier SNPs for ancient data analysis represented on both references (LipY764 and chrY)***

Based on ‘horseYtree.vs1’ (see above) we extracted 1,031 SNPs (not including C > T and G > A transitions [23]) as ‘identifier variants’ for the mjHGs and their coordinates and orientation were determined on chrY. For this step, -/+ 30bp flanking regions of each SNP were extracted for from LipY764 reference as a fasta file which was then mapped to the chrY reference with bwa (ver. 0.7.17) [7] aln function in default mode. 232 identifier SNPs could be located on chrY whereas 114 were on the forward and 118 on the reverse strand. The 232 identifiers are marked in Supplementary Table 4 (Column GK-GN), together with coordinates and orientation on chrY.

### Genotyping identifier variants in ancient samples

The 232 identifier SNPs were genotyped in the 282 ancient samples with gatk (version 4.1.4.1) haplotype caller [24] in ploidy 1 mode providing the coordinates of the reference they were mapped to. Out of the 232 identifier SNPs, 105 SNPs (Supplementary Table 4 Column GN) could be genotyped on both references and out of those 96 were identifiers for four major branches: 18 p_identifiers, 47 d_identifiers, 22 db_identifiers and 9 da1_identifiers. The binary genotyping outputs were converted to ancestral and derived states based on the alleles’ status defined in the network classification step for ‘horseYtree.vs1’. For each sample, the number of genotyped positions was calculated, and only samples having at least 48 genotyped positions (50 %) were further analyzed (n= 169, Supplementary Table 5 Column P).

### Clustering

Samples were clustered based on the number of derived states at identifier variants. If a sample carried the derived allele in at least half of the identifier variants in a branch (9 for p, 23 for d, 11 for db and 4 for da1) and no ancestral allele the sample was located into that cluster. Exceptionally, two samples (SAGS27_Ira_3052BP and Bi5x1_Rus_2575BP) that carried only two/three derived states at da1 identifiers were clustered in da1.

Some samples showed both, ancestral and derived states for identifier SNPs on a branch, meaning their HTs split off on that branch with their private variation not yet ascertained from modern data. Those HGs, only represented by ancient samples were formed by Greek letters of the corresponding HG name in modern samples followed by a ‘*’ (for instance, π* for p haplogroup).

Samples passing the filtering thresholds but showing ancestral alleles on the majority of genotyped positions (a maximum of 3 derived states in total (error lot)), were placed to the root (ρο). Six out of the 169 samples did not fit any of the above-mentioned categories and were not placed.

The 84 samples clustering in da1 were also checked for identifier SNPs of mjHGs in da1. Nine SNPs identifying further mjHGs in da1 (da1_N, da1_Ub-e, Ub-i, da1_Um, da3_Y, and daC) were used. The allelic state and read depth were checked and 53 samples passed our threshold, as they were genotyped in at least 3 (out of 9) positions. Those 53 samples were used for da1 mjHG clustering. If a sample carried the derived state for identifier/s of a HG it was clustered in that HG. Because of the low number of identifiers on each branch (one to three) the number of reads showing the derived state was also considered. Samples having only one read support for a derived state were clustered conservatively in the basal node da1. For the single sample showing the pattern of 1 derived and 2 ancestral Ub-e identifiers, a new HG (δα1_Υβ-ε*) was defined.

The visualization of clustering and locating samples on a map was created in R (ver.3.6.1) [19].

***Supplementary Information References***

1. Felkel, S.; Vogl, C.; Rigler, D.; Dobretsberger, V.; Chowdhary, B.P.; Distl, O.; Fries, R.; Jagannathan, V.; Janečka, J.E.; Leeb, T.; et al. The horse Y chromosome as an informative marker for tracing sire lines. *Sci. Rep.* **2019**, *9*, 1–12, doi:10.1038/s41598-019-42640-w.

2. Felkel, S.; Vogl, C.; Rigler, D.; Jagannathan, V.; Leeb, T.; Fries, R.; Neuditschko, M.; Rieder, S.; Velie, B.D.; Lindgren, G.; et al. Asian horses deepen the MSY phylogeny. *Anim. Genet.* **2018**, *49*, 90–93, doi:10.1111/age.12635.

3. Han, H.; Wallner, B.; Rigler, D.; MacHugh, D.E.; Manglai, D.; Hill, E.W. Chinese Mongolian horses may retain early domestic male genetic lineages yet to be discovered. *Anim. Genet.* **2019**, *50*, 399–402, doi:10.1111/age.12780.

4. Hammer, M.F. A nomenclature system for the tree of human Y-Chromosomal binary haplogroups. *Genome Res.* **2002**, *12*, 339–348, doi:10.1101/gr.217602.

5. Schubert, M.; Lindgreen, S.; Orlando, L. AdapterRemoval v2: Rapid adapter trimming, identification, and read merging. *BMC Res. Notes* **2016**, *9*, 1–7, doi:10.1186/s13104-016-1900-2.

6. Gómez-Sánchez, D.; Schlötterer, C. ReadTools: A universal toolkit for handling sequence data from different sequencing platforms. *Mol. Ecol. Resour.* **2018**, *18*, 676–680, doi:10.1111/1755-0998.12741.

7. Li, H.; Durbin, R. Fast and accurate short read alignment with Burrows-Wheeler transform. *Bioinformatics* **2009**, *25*, 1754–1760, doi:10.1093/bioinformatics/btp324.

8. Li, H.; Handsaker, B.; Wysoker, A.; Fennell, T.; Ruan, J.; Homer, N.; Marth, G.T.; Abecasis, G.R.; Durbin, R. The Sequence Alignment/Map format and SAMtools. *Bioinformatics* **2009**, *25*, 2078–2079, doi:10.1093/bioinformatics/btp352.

9. Garrison, E.; Marth, G. Haplotype-based variant detection from short-read sequencing. *arXiv Prepr.* **2012**, *1207.3907*, 1–9.

10. Danecek, P.; Auton, A.; Abecasis, G.; Albers, C.A.; Banks, E.; DePristo, M.A.; Handsaker, R.E.; Lunter, G.; Marth, G.T.; Sherry, S.T.; et al. The variant call format and VCFtools. *Bioinformatics* **2011**, *27*, 2156–2158, doi:10.1093/bioinformatics/btr330.

11. Li, H. A statistical framework for SNP calling, mutation discovery, association mapping and population genetical parameter estimation from sequencing data. *Bioinformatics* **2011**, *27*, 2987–2993, doi:10.1093/bioinformatics/btr509.

12. Van Rossum, O.; Drake, F.L. Python 3 Reference Manual. 2009.

13. Remer, V.; Bozlak, E.; Felkel, S.; Radovic, L.; Rigler, D.; Grilz-Seger, G.; Stefaniuk-Szmukier, M.; Bugno-Poniewierska, M.; Brooks, S.; Miller, D.C.; et al. Y-Chromosomal Insights into Breeding History and Sire Line Genealogies of Arabian Horses. *Genes (Basel).* **2022**, *13*, doi:10.3390/genes13020229.

14. Thorvaldsdóttir, H.; Robinson, J.T.; Mesirov, J.P. Integrative Genomics Viewer (IGV): High-performance genomics data visualization and exploration. *Brief. Bioinform.* **2013**, *14*, 178–192, doi:10.1093/bib/bbs017.

15. Stecher, G.; Tamura, K.; Kumar, S. Molecular evolutionary genetics analysis (MEGA) for macOS. *Mol. Biol. Evol.* **2020**, *37*, 1237–1239, doi:10.1093/molbev/msz312.

16. FigTree Available online: http://tree.bio.ed.ac.uk/software/figtree/.

17. Excoffier, L.; Laval, G.; Schneider, S. Arlequin (version 3.0): An integrated software package for population genetics data analysis. *Evol. Bioinforma.* **2005**, *1*, 117693430500100, doi:10.1177/117693430500100003.

18. Watterson, G.A. On the number of segregating sites in genetical models without recombination. *Theor Popul Biol.* **1975**, *7*, 256–76.

19. R Core Team R: A language and environment for statistical computing 2017.

20. Bandelt, H.J.; Forster, P.; Röhl, A. Median-joining networks for inferring intraspecific phylogenies. *Mol. Biol. Evol.* **1999**, *16*, 37–48, doi:10.1093/oxfordjournals.molbev.a026036.

21. Wallner, B.; Palmieri, N.; Vogl, C.; Rigler, D.; Bozlak, E.; Druml, T.; Jagannathan, V.; Leeb, T.; Fries, R.; Tetens, J.; et al. Y Chromosome Uncovers the Recent Oriental Origin of Modern Stallions. *Curr. Biol.* **2017**, *27*, 2029-2035.e5, doi:10.1016/j.cub.2017.05.086.

22. Gaunitz, C.; Fages, A.; Hanghøj, K.; Albrechtsen, A.; Khan, N.; Schubert, M.; Seguin-Orlando, A.; Owens, I.J.; Felkel, S.; Bignon-Lau, O.; et al. Ancient genomes revisit the ancestry of domestic and Przewalski’s horses. *Science (80-. ).* **2018**, *360*, 111–114, doi:10.1126/science.aao3297.

23. Dabney, J.; Meyer, M.; Pääbo, S. Ancient DNA damage. *Cold Spring Harb. Perspect. Biol.* **2013**, *5*, 1–7, doi:10.1101/cshperspect.a012567.

24. McKenna, A.; Hanna, M.; Banks, E.; Sivachenko, A.; Cibulskis, K.; Kernytsky, A.; Garimella, K.; Altshuler, D.; Gabriel, S.; Daly, M.; et al. The Genome Analysis Toolkit: a MapReduce framework for analyzing next-generation DNA sequencing data. *Genome Res.* **2010**, *20*, 1297–1303, doi:doi.org/10.1101/gr.107524.110.
